# High-efficiency transduction of spinal cord motor neurons by intrauterine delivery of integration-deficient lentiviral vectors

**DOI:** 10.1016/j.jconrel.2017.12.029

**Published:** 2018-03-10

**Authors:** Sherif G. Ahmed, Simon N. Waddington, Maria Gabriela Boza-Morán, Rafael J. Yáñez-Muñoz

**Affiliations:** a AGCTlab.org, Centre for Biomedical Sciences, School of Biological Sciences, Royal Holloway, University of London, Egham TW20 0EX, UK; b Department of Pharmacology and Toxicology, Faculty of Pharmacy, Beni-Suef University, Egypt; c The Institute for Women's Health, University College London, London, UK; d MRC Antiviral Gene Therapy Research Unit, Faculty of Health Sciences, University of the Witwatersrand, Johannesburg, South Africa

**Keywords:** Integration-deficient lentiviral vectors (IDLV), Spinal cord, *In utero* gene delivery, Pseudotyping

## Abstract

Integration-deficient lentiviral vectors (IDLVs) are promising gene delivery tools that retain the high transduction efficiency of standard lentiviral vectors, yet fail to integrate as proviruses and are instead converted into episomal circles. These episomes are metabolically stable and support long-term expression of transgenes in non-dividing cells, exhibiting a decreased risk of insertional mutagenesis. We have embarked on an extensive study to compare the transduction efficiency of IDLVs pseudotyped with different envelopes (vesicular stomatitis, Rabies, Mokola and Ross River viral envelopes) and self-complementary adeno-associated viral vectors, serotype-9 (scAAV-9) in spinal cord tissues after intraspinal injection of mouse embryos (E16). Our results indicate that IDLVs can transduce motor neurons (MNs) at extremely high efficiency regardless of the envelope pseudotype while scAAV9 mediates gene delivery to ~ 40% of spinal cord motor neurons, with other non-neuronal cells also transduced. Long-term expression studies revealed stable gene expression at 7 months post-injection. Taken together, the results of this study indicate that IDLVs may be efficient tools for *in utero* cord transduction in therapeutic strategies such as for treatment of inherited early childhood neurodegenerative diseases.

## Graphical abstract

**Figure:**
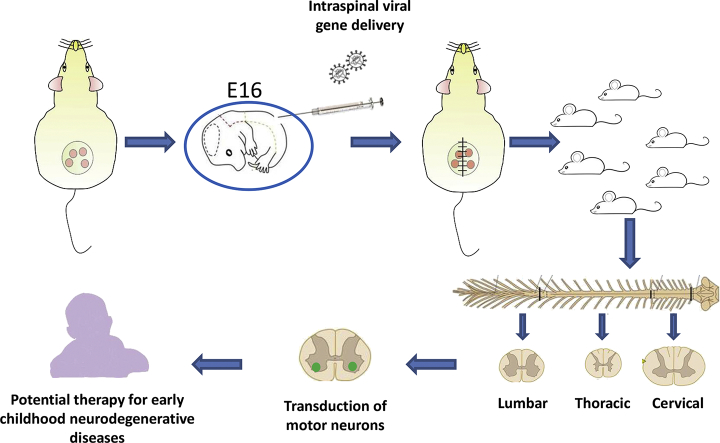

## 1 Introduction

A key factor in the success of gene therapy is the development of delivery systems that are capable of efficient gene transfer without causing pathogenic effects. Lentiviral vectors, engineered forms of retroviruses from the genus *Lentivirus*, are promising tools for gene therapy because they transduce dividing and quiescent cells efficiently, their tropism can be changed by replacing the natural envelope of lentiviruses with a heterologous envelope (referred to as pseudotyping) and they lead to strong and long-lasting expression of transgenes [1]. However, during the transduction process lentiviral vectors integrate their genome in the host cell DNA, which can lead to insertional mutagenesis. Retroviral vector-mediated insertional mutagenesis has become clinically relevant, having led to several cases of T-cell leukemia in the clinical trials of gene therapy for X-linked severe combined immunodeficiency (SCID-X1) and chronic granulomatous disease (CGD) [2], [3]. Albeit lower, some risk of insertional mutagenesis is also present using integrating lentiviral vectors [4]. Engineering of self-inactivating (SIN) lentiviral vectors by deletion of the U3 region in the 3′-LTR is considered to minimize the probability of interactions between the transfer vector elements and the host genome and consequently the incidence of insertional mutagenesis-related events in transduced cells [5]. The non-transcribing LTR in these vectors is not able to drive the expression of cellular oncogenes that might be present in the vicinity of the integration site [6], [7], [8]. Studies with SIN lentiviral vectors containing enhancer-less internal promoters resulted in safety improvements in different preclinical studies [9], [10], [11], [12], [13].

The use of integration-deficient lentiviral vectors (IDLVs) is another strategy that may overcome the insertional mutagenesis risks. These vectors have been developed by introducing class I mutations, which impair the catalytic activity of integrase but do not affect the amount of vector DNA produced, into the integrase gene [14]. Failing integration, the viral DNA of these mutants is converted into episomal circles by nuclear enzymes. The viral episomes are diluted out in proliferating tissues while they are retained and metabolically stable in quiescent cells. Therefore, IDLVs could be an ideal delivery vector for transduction of post-mitotic central nervous system (CNS) cells such as neurons. Yáñez-Muñoz et al. demonstrated that HIV-1-based IDLVs produced with a D64V integrase mutation efficiently transduced ocular and brain tissues in rodents, in addition to exhibiting an expression level of *eGFP* in the eye comparable with vectors containing wild-type integrase [15], [16]. Gene delivery and expression in striatum using similar IDLVs but with the 262RRK to AAH integrase mutation were demonstrated by Philippe et al. [17]. Rahim et al. showed that IDLVs resulted in efficient long-term expression in the CNS following *in utero* delivery [18]. More recently, Peluffo et al. explored *in vivo* transduction of spinal cord by IDLVs in adult rodents. In both mice and rats, intraparenchymal injection of IDLV-*eGFP* in the ventral horn of the spinal cord led to significant transduction of motor neurons (MNs) around the injection site [19]. IDLVs expressing glial derived neurotrophic factor have been also shown to induce neuroprotection in a rat model of Parkinson's disease [20].

The CNS can be affected by a number of genetic diseases that start developing before birth [21]. In such cases, *in utero* gene transfer seems a promising technology. Widespread gene delivery to the nervous system is feasible using fetal delivery and may offer several advantages [22], [23]. Firstly, the relatively smaller size of the fetus allows exposure of wider areas to the high concentration of vector that may be required for therapeutic effect. Secondly, a fetal therapy approach may prevent, reverse or ameliorate the onset of pathological changes that could be irreversible later in development. Thirdly, because of the immature immune system and unlikely exposure to the archetypal viruses from which viral vectors have been developed, fetal delivery may lead to immune tolerance to injected viral vector and the transgenic product, unlike in the adults [24].

Here we demonstrate that intrauterine injection of various pseudotyped IDLVs achieves efficient and persistent gene expression in motor neurons within the mouse spinal cord. We also show that IDLVs produce better and more selective transduction of motor neurons in comparison to scAAV9, which displayed significant non-neuronal transduction. These promising results could guide the choice of vector to be applied for *in utero* delivery, depending on the transduction pattern most likely to result in therapeutic benefit.

## 2 Materials and methods

### 2.1 Production of IDLVs

Third-generation self-inactivating HIV-1-based vectors were produced by transient co-transfection of four plasmids in human embryonic kidney 293 T cells as previously described [25]. The transfer plasmid was pRRLsin_PPT_CMV_*GFP*pre, a self-inactivating lentiviral construct containing a CMV promoter driving *eGFP* expression, followed by the Woodchuck hepatitis virus post-transcriptional regulatory element (WPRE) [26]. The packaging plasmids were pRSV-rev and pMDLg/pRREintD64V [27]. Vectors were pseudotyped with either vesicular stomatitis virus G glycoprotein (VSV-G; plasmid pMD2.G), rabies virus envelope glycoprotein (plasmid pHCMV.rabiesG, kindly provided by M Sena-Esteves, University of Massachusetts), Ross-River virus envelope (plasmid pRRV_ENV, kind gift of C. Lundberg, Lund University) or Mokola virus envelope (plasmid pHCMV-Mokola-G, from M. Sena-Esteves). Vectors were concentrated by ultracentrifugation and titrated as described [28].

### 2.2 AAV production

Recombinant self-complementary AAV vectors of capsid serotype 9 (scAAV9) were produced using a triple-transfection calcium phosphate method in HEK293 cells. Plasmids p5E18-VD2/9 (packaging plasmid), pAdDeltaF6 (adenovirus helper plasmid) and transfer plasmid pscAAVCAGe*GFP* (encoding *eGFP* driven by the cytomegalovirus enhancer/chicken beta actin CAG promoter) were used to generate scAAV9.CAG.*GFP*. At 72 h post transfection, cell cultures were centrifuged and vector in the supernatant was recovered by over-night precipitation. The precipitated supernatant and cell pellet resuspended in lysis buffer were mixed and subjected to three freeze/thaw cycles for 10 min at 37 °C and then 30 min at − 80 °C. Benzonase was added at a final concentration of 50 U/ml, incubating for 30 min at 37 °C, followed by 25 min centrifugation at 3360 ×* g* at RT and further filtration through a 0.45 μm pore size filter. The clarified lysate was loaded onto a modified discontinuous iodixanol gradient followed by column chromatography. Purified vectors were titrated by estimating number of genome copies (GC) by quantitative PCR and dot-blot [29].

### 2.3 *In utero* injections and histology

All animal procedures were carried out under the provisions of the UK Animals (Scientific Procedures) Act 1986. Pregnant CD1 mice at 16 days gestation were used. Intrauterine surgery was performed as previously described [18]. Under isoflurane anaesthesia, the uteri were exposed through a midline laparotomy. *Trans*-uterine injections of IDLVs with 2.03E + 09 GC/ml and scAAV9 with 6.42E + 10 GC/ml into the spinal cord (2 μl), the anterior horn of the lateral ventricle on the left side of the brain (5 μl) or the superficial temporal vein (20 μl) of the fetuses were given using a 34-gauge needle (Hamilton UK). All the fetuses were injected. The laparotomy was repaired in two stages using interrupted 6-0 silk sutures. Dams were allowed to recover in a warm cage. Mice were euthanized at the indicated times post-injection with isofluorane overdose and spinal cords were removed and placed in PBS for visualizing eGFP fluorescence microscopically. Given the intensity of eGFP fluorescence achieved, cords were occasionally discarded as injection fails at this stage if no fluorescence was observed; this technical failure rate was estimated at around 25%. The cords were then immersed in 4% PFA overnight for fixation. They were subsequently transferred to 20% sucrose for 24–48 h for cryoprotection, embedded in OCT medium and snap-frozen in liquid nitrogen. Twenty-micrometer coronal sections were cut on a cryostat, mounted on Super Frost slides (BDH) and stored at − 80 °C for subsequent analysis.

### 2.4 Immunohistochemistry

Cryosection slides were removed from storage at − 80 °C and left at RT for 5 min to air dry. The slides were washed in 1 × PBS for 5 min three times, and permeabilized with 1 × PBS with 0.05% (v/v) Tween for 10 min. They were incubated with 2% bovine serum albumin for 1 h to block non-specific binding, washed with 1 × PBS and incubated overnight at 4 °C with primary antibody diluted in PBS. The slides were washed 3 times for 5 min with PBS and incubated for 1 h with secondary antibody conjugated with fluourochrome, diluted in 1 × PBS. The sections were washed 3 times for 5 min in 1 × PBS and mounted with Vectashield containing DAPI (VectorLabs) for nuclear staining. They were stored at 4 °C or examined directly under a Zeiss Axio Observer.Z1 fluorescence microscope (Zeiss, UK). Primary antibodies: goat anti-ChAT (Millipore, UK, 1:50, Cat. AB144P), mouse anti-NeuN (Millipore, UK 1:500, Cat. MAB377), rabbit anti-GFAP (DAKO, UK, 1:500, Cat. Z033429-2), rabbit anti-IBA-1 (WAKO, Osaka, Japan; Cat. 019–19,741, 1:1000), mouse anti-APC (Calbiochem, Darmstadt, Germany; Cat. OP80 Ab-7, 1:500), rabbit anti-e*GFP* (Invitrogen, UK, 1:1000, Cat. A-11122) and mouse anti-e*GFP* (UC Davis/NINDS NeuroLab Facility, clone N86/38, 1:1000) were used. Donkey anti goat Alexa 555 (Invitrogen, UK, 1:1000, Cat. A-21432), goat anti mouse Alexa 555 (Invitrogen, UK, 1:1000, Cat. A-21424), goat anti rabbit Alexa 555 (Invitrogen, UK, 1:1000, Cat. A32732) and goat anti rabbit Alexa 488 (Invitrogen, UK, 1:1000, Cat. A-11034) were used as secondary antibodies.

### 2.5 Cell counts

Coronal spinal cord sections showing the entire ventral horn at the relevant level were processed for double immunohistochemistry for eGFP and ChAT, NeuN or GFAP and analyzed with 20 × and 40 × magnification using a Zeiss Axio Observer.Z1 fluorescence microscope (Zeiss, UK) for accurate cell-type identification. For this, the spinal cord was divided into cervical, thoracic and lumbar segments, based on the vertebral levels. Segments were processed into 20 micrometer-thick sections and these were stored, to carry out assessments of gene expression. Approximately 36 sections per segment per animal were processed for immunohistochemistry. Approximately 1 in every 6 consecutive sections was analyzed for each cell type marker. Images were captured and used for manual quantification of percentages of *in vivo* neuronal transduction. The percent of eGFP-positive motor neurons or neurons was averaged for each segment. Three mice were analyzed per vector injected (*N* = 3).

### 2.6 Statistical analysis

Statistical analyses were performed using Graph Pad Prism software. Means were represented with s.e.m. One-way analysis of variance was used to assess for significant differences among groups. Student's *t*-tests were performed to compare P10 *versus* 7 months post-injection using a 95% confidence level.

## 3 Results

### 3.1 Optimisation of the route of injection

**Fig. 1:**
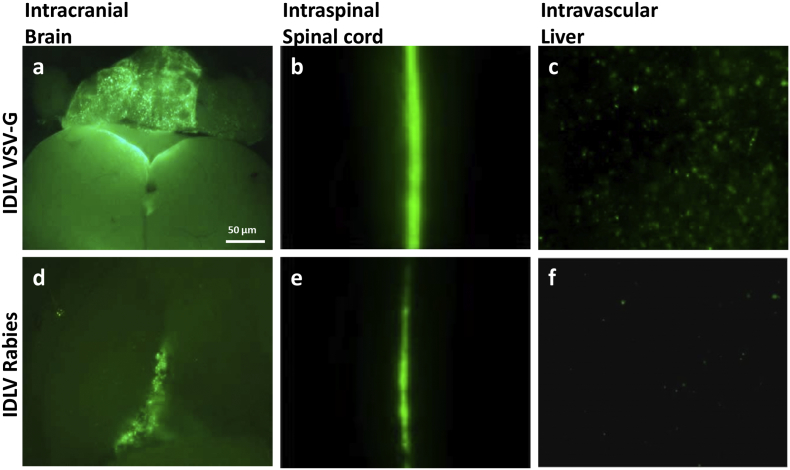
Strong *eGFP* expression after *in utero* injection with IDLV-VSV-G and IDLV-Rabies. *eGFP* expression is mostly localised to the tissues where the viral vectors were injected. Intracranial and intraspinal injection of either viral vector showed localised *eGFP* expression in the brain (a, d) and spinal cord (b, e), respectively. Intravascular injection showed *eGFP* expression in the liver, particularly for the IDLV-VSV-G (c) but not so much for the rabies pseudotyped IDLV (f). Lack of eGFP fluorescence in uninjected and DMEM-injected cords is demonstrated in Supplementary Fig. 4a and b, respectively.

A pilot experiment was performed to identify the optimal fetal route of injection for gene delivery to the spinal cord. IDLVs carrying *eGFP* under transcriptional control of the CMV promoter were produced using either VSV-G or Rabies envelope proteins. These were injected into E16 fetal mice by intraspinal, intracranial, or intravascular routes. Tissues were harvested ten days after birth (P10). Intraspinal injection showed robust *eGFP* fluorescence along the entire spinal cord, with expression additionally observed in spinal ganglia; IDLV-Rabies also produced *GFP* fluorescence in the intercostal nerves (data not shown). Under UV illumination *eGFP* expression was sufficiently strong to be visible to the naked eye in whole dissected spinal cords. Intracranial injection resulted in expression which was restricted to the brain. Intravenous injection resulted in e*GFP* expression only in the liver. IDLV-VSV-G showed stronger *eGFP* expression than IDLV-Rabies in the brain and liver after intracranial and intravascular injection respectively. Intraspinal injection of both IDLV-VSV-G and IDLV-Rabies showed similar intensity of *eGFP* expression (Fig. 1). It was therefore concluded that intraspinal injections, through which the entire cord is bathed in vector solution at the time of injection, were most appropriate for cord transduction.

A more detailed assessment of transduction by IDLVs containing CMV-*eGFP* was carried out with vectors pseudotyped with VSV-G, Rabies, Mokola or Ross River virus envelope proteins. For comparative purposes we also used self-complementary AAV serotype 9 (scAAV9), in which the expression of *eGFP* was driven by the cytomegalovirus enhancer/chicken beta-actin (CAG) promoter. All viral vectors were injected intraspinally into E16 mice fetuses, and samples harvested at P10.

**Fig. 2:**
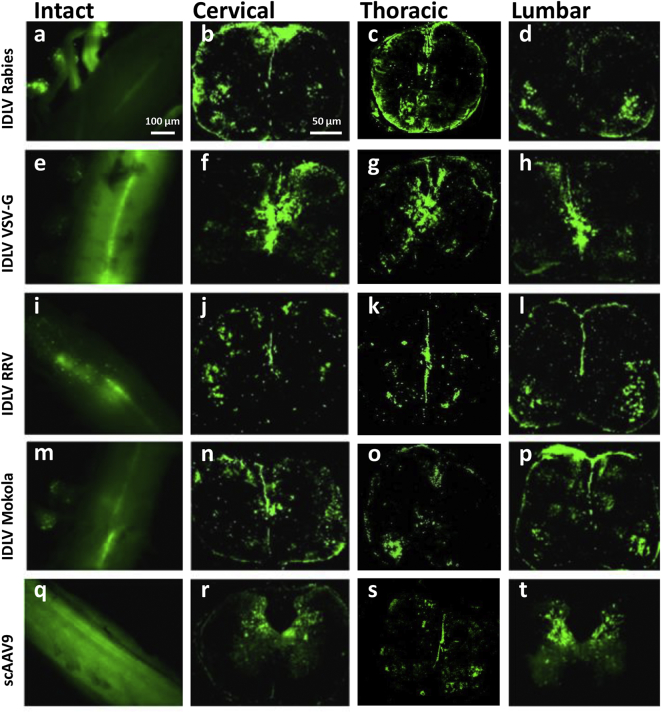
Broad and efficient transduction of the spinal cord with pseudotyped IDLVs (CMV-*eGFP*) and scAAV9 (CAG-*eGFP*) after intraspinal delivery *in utero*. *eGFP* expression can be observed in the entire cord of samples harvested at P10 in whole cord (“intact”) and coronal sections at cervical, thoracic and lumbar levels (dorsal region is at the top and ventral region is at the bottom in all coronal sections).

Widespread *eGFP* fluorescence was confirmed in whole spinal cord and ganglia of animals injected with IDLVs (Fig. 2a, e, i, m, q). Coronal sections of the cord were analyzed for *eGFP* fluorescence. The results were similar for all IDLVs regardless of pseudotype, showing significant transduction at cervical, thoracic and lumbar levels (Fig. 2).

Transduction with scAAV9 also showed extensive transgenic expression in the spinal cord at dissection. When analyzed in coronal sections, eGFP fluorescence in the cord parenchyma seemed more extensive than with IDLVs (Fig. 2r, s, t).

### 3.2 Neural cell-type-specific transduction with IDLVs

**Fig. 3:**
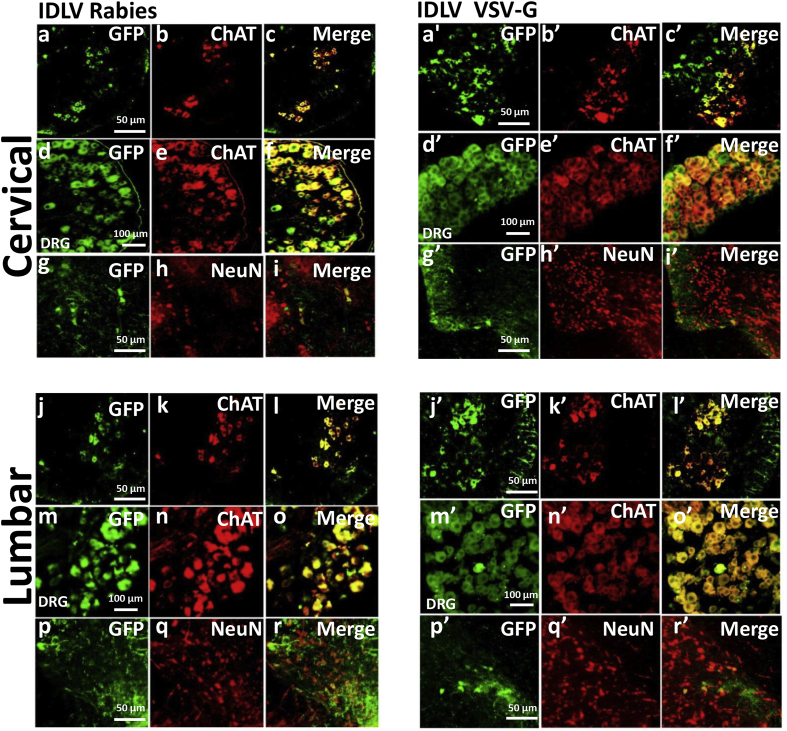
Strong neuronal expression of *eGFP* after E16 intraspinal IDLV delivery. Transduction of motor neurons and neurons following *in utero* delivery of the indicated IDLV CMV-*eGFP* vectors was assessed by co-staining with relevant markers. Extensive transduction of ChAT + motor neurons in the ventral region of coronal cord sections and DRG neuron cell bodies (middle row in all panels) was observed. *eGFP* expression was also visible in variable levels of NeuN + neurons.

To assess cell-type specificity of transgenic expression after *in utero* delivery of IDLVs or scAAV9, transduction efficiency in the major cell types in the CNS (motor neurons, neurons, astrocytes, oligodendrocytes and microglia) was evaluated histologically. Co-staining for markers of the relevant cell types revealed motor neurons to be the most efficiently and broadly transduced. ChAT-positive motor neurons throughout the entire spinal cord strongly expressed *eGFP* in mice injected with IDLVs regardless of the type of envelope used; (VSV-G and rabies, Fig. 3 and Supplementary Fig. 1; Mokola and RRV, Supplementary Figs. 1 and 2). Motor neuron transduction appeared to be complete (Table 1). Dorsal root ganglia neurons also contained a ChAT + population which showed strong and extensive *eGFP* expression at all levels of the cord. Estimates of transduction of NeuN + neurons across cord segments revealed higher transduction by the rabies pseudotyped IDLV (~ 35%) than VSV-G (~ 15%), RRV (~ 13%) and Mokola IDLVs (~ 11%; Table 1). Co-staining for astrocytes (GFAP +), oligodendrocytes (APC +) or microglia (Iba1 +) did not reveal significant transduction or activation of any of these populations in sections from mice injected with the various IDLVs (Supplementary Figs. 3 and 4).

**Table 1:** Frequency of transduced cells by cell type, vector, and spinal cord region in samples harvested at P10.

	IDLV-CMV-GFP-VSV-G				IDLV-CMV-GFP-Rabies				IDLV-CMV-GFP-RRV			
	N	GFP +/ChAT +	Total ChAT +	%	N	GFP +/ChAT +	Total ChAT +	%	N	GFP +/ChAT +	Total ChAT +	%
Cervical	3	259	259	100	3	234	234	100	3	243	243	100
Thoracic	3	146	146	100	3	151	151	100	3	137	137	100
Lumbar	3	264	264	100	3	223	223	100	3	237	237	100

	N	GFP +/NeuN +	Total NeuN +	%	N	GFP +/NeuN +	Total NeuN +	%	N	GFP +/NeuN +	Total NeuN +	%
Cervical	3	74	463	15.9 ± 6	3	163	455	35.8 ± 3	3	66	477	13.8 ± 4
Thoracic	3	61	441	13.8 ± 5	3	151	413	36.5 ± 4	3	51	411	12.4 ± 3
Lumbar	3	73	487	14.9 ± 6	3	156	447	34.8 ± 7	3	59	461	12.7 ± 4


	IDLV-CMV-GFP-Mokola				scAAV9-CAG-GFP			
	N	GFP +/ChAT +	Total ChAT +	%	N	GFP +/ChAT +	Total ChAT +	%
Cervical	3	262	262	100	3	97	271	35.7 ± 7
Thoracic	3	133	133	100	3	86	263	32.6 ± 4
Lumbar	3	243	243	100	3	101	266	37.9 ± 4

	N	GFP +/NeuN +	Total NeuN +	%	N	GFP +/NeuN +	Total NeuN +	%
Cervical	3	46	419	10.9 ± 7	3	69	437	15.7 ± 5
Thoracic	3	43	403	10.6 ± 6	3	55	410	13.4 ± 3
Lumbar	3	51	431	11.8 ± 4	3	67	453	14.7 ± 5

### 3.3 *In utero* injection of scAAV9 in the spinal cord shows different tropism

**Fig. 4:**
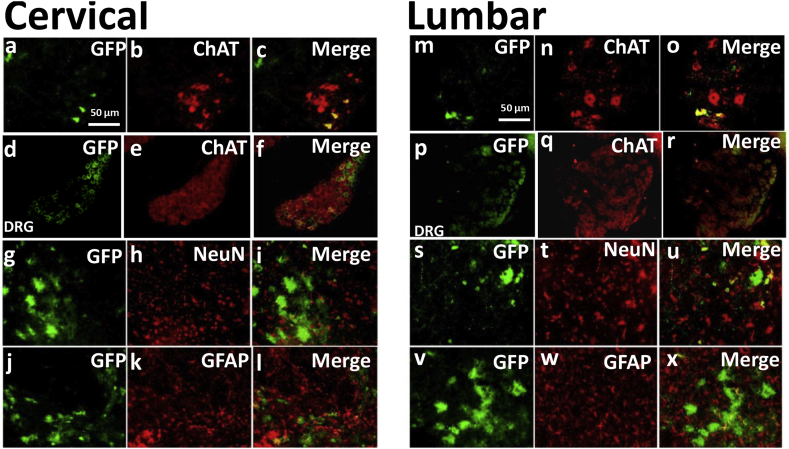
Transduction pattern following intrauterine delivery of scAAV9 CAG-*eGFP*. Cervical and lumbar sections of the harvested P10 cords show partial transduction of ChAT + motor neurons (a, b, c, m, n, o) and DRG neurons (d, e, f, p, q, r), and NeuN + neurons (g, h, i, s, t, u). GFAP + astrocytes often co-localised with eGFP fluorescence (j, k, l, v, w, x).

Spinal cords of mice injected with scAAV9 CAG-*eGFP* showed different vector tropism and transduction efficiency compared to IDLV injections. The number of *eGFP* + motor neurons was significantly (*p* < 0.001) lower: an average of 35.4% ChAT + motor neurons showed eGFP fluorescence (Table 1 & Fig. 4a, b, c, m, n, o). NeuN + neurons showed *eGFP* expression in 14.6% of cases (Table 1 & Fig. 4g, h, i, s, t, u), statistically lower than IDLV-Rabies (p < 0.001). Unlike with IDLVs, GFAP + astrocytes were often transduced with scAAV9 and showed strong *eGFP* expression (Fig. 4j, k, l, v, w, x). No transduction or activation of Iba1 microglia were observed (Supplementary Fig. 4i, j, k).

### 3.4 Long-term expression from IDLVs and scAAV9

**Fig. 5:**
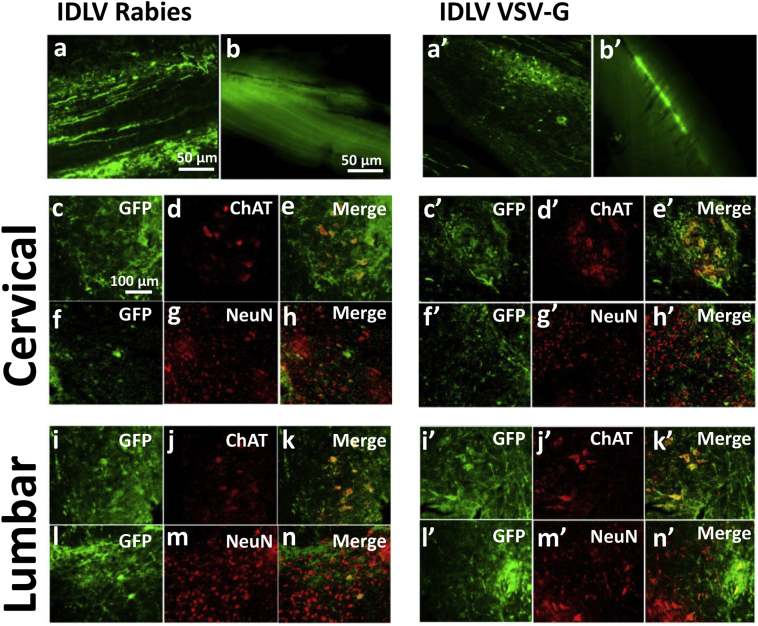
Immunofluorescence studies confirm long-term motor neuron-specific gene delivery in the spinal cord following *in utero* treatment with IDLVs. Long-term gene expression was confirmed by *eGFP* fluorescence and co-staining of sections of spinal cords from mice harvested 7 months after *in utero* administration of IDLVs pseudotyped with rabies (left panel) or VSV-G (right panel). Strong *eGFP* expression can be seen on longitudinal sections (a, a′) and along whole cords (b, b′). ChAT + cells show *eGFP* co-expression in coronal cervical (c, d, e, c′, d′, e′) and lumbar (i, j, k, i′, j′, k′) sections of mice injected with either vector. *eGFP* co-localization was also observed in some neurons stained with NeuN antibody (f, g, h, l, m, n, f′, g′, h′, l′, m′, n′).

**Fig. 6:**
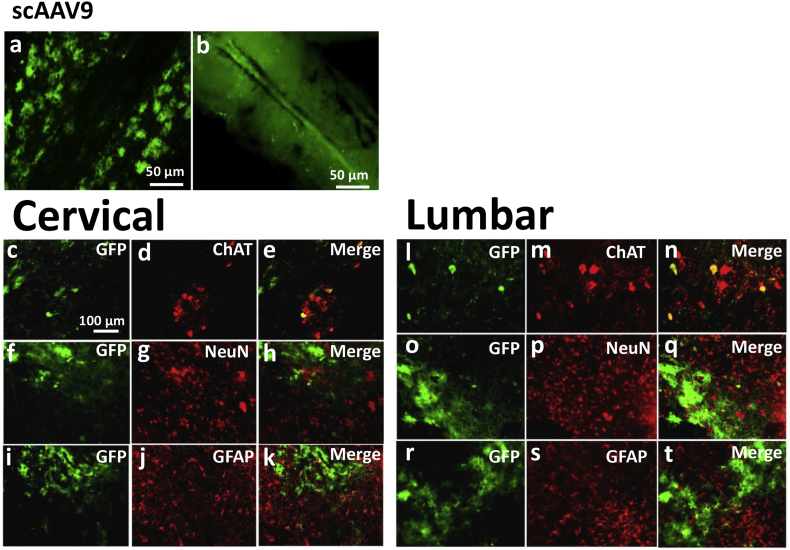
Long-term expression in motor neurons, neurons and astrocytes after E16 intraspinal injection with scAAV9. Longitudinal sections (a) and whole spinal cord (b) demonstrated strong *eGFP* expression 7 months post-injection. Co-stained sections revealed same transduction pattern as at the P10 time-point. Partial ChAT + motor neuron staining colocalized with *eGFP* (c, d, e, l, m, n). Limited neuron (f, g, h, o, p, q) and astrocyte (i, j, k, r, s, t) transduction were observed through co-localization of *eGFP* and NeuN or GFAP.

To assess long-term transgene expression after *in utero* intraspinal injection, mice were treated with VSV-G or rabies-pseudotyped IDLVs, or scAAV9, and euthanised at 7 months. Comparing with the P10 time-point, *eGFP* expression was stable, with no obvious reduction in the frequency of e*GFP* + cells over time (Table 2). *eGFP* expression was again sufficiently strong for fluorescence to be visible to the naked eye in whole dissected cords. The distinct transduction efficiency and tropism of pseudotyped IDLVs and scAAV9 were maintained at P10 and the 7-month time-points (Fig. 5, Fig. 6, and Table 2).

**Table 2:** Frequency of transduced cells by cell type, vector, and spinal cord region in samples harvested at postnatal month 7.

	IDLV-CMV-GFP-VSV-G				IDLV-CMV-GFP-Rabies				scAAV9-CAG-GFP			
	N	GFP +/ChAT +	Total ChAT +	%	N	GFP +/ChAT +	Total ChAT +	%	N	GFP +/ChAT +	Total ChAT +	%
Cervical	3	243	243	100	3	222	222	100	3	93	266	34.9 ± 7
Thoracic	3	133	133	100	3	131	131	100	3	71	220	32.2 ± 4
Lumbar	3	259	259	100	3	227	227	100	3	107	264	40.5 ± 4

	N	GFP +/NeuN +	Total NeuN +	%	N	GFP +/NeuN +	Total NeuN +	%	N	GFP +/NeuN +	Total NeuN +	%
Cervical	3	63	455	13.8 ± 6	3	166	461	36.1 ± 3	3	67	423	15.8 ± 5
Thoracic	3	71	443	16.1 ± 5	3	148	417	35.4 ± 4	3	49	395	12.4 ± 3
Lumbar	3	83	496	16.7 ± 6	3	149	441	34.7 ± 7	3	77	463	16.6 ± 5

## 4 Discussion and conclusion

Delivery of viral vectors to the fetal organs and tissues is an important strategy in studies of diseases of the central and peripheral nervous system. From a therapeutic viewpoint, *in utero* gene therapy is an attractive principle in which fetal delivery of transgenes may prevent the pathology of early-onset diseases, induce tolerance against an expressed therapeutic protein and increase likelihood of widespread transduction [30]. Prenatal gene therapy has been successfully used in a number of animal disease models [31], [32] and may serve as a platform for novel strategies to treat a wide variety of neurological disorders. Lentiviral vectors, with their relatively high payload and ability to transduce quiescent and dividing cell populations, are an important tool among the available vector systems [33].

Targeted delivery and expression of therapeutic genes into specific cell populations is a crucial need in the field of gene therapy, notably in the CNS. The main methods that have been used to achieve cell-type specific lentiviral transduction are: route of delivery, vector pseudotyping, cell-type specific promoters and vector detargeting. Delivery route and timing have been extensively explored both in adults and perinatally with a variety vectors, including standard lentiviral vectors and IDLVs [34]. Pseudotyping lentiviral vectors with different natural envelope glycoproteins has been shown to alter the tropism towards glial or neuronal cells [35]. More recently, engineering of chimeric envelope proteins has allowed lentiviral targeting, including in the CNS [36], [37]. Promoters such as those for enolase and synapsin I have been used for neuronal-specific expression, and the human GFAP promoter for glial-specific expression [38], [39]. Finally, lentiviral vector detargeting using miRNAs has been an effective way to prevent expression of transgenes in specific cell-types [40], including neurons [41].

Based upon preliminary proof-of-concept injections of vector into the spinal cord of fetal mice [34], we have further explored route of delivery and pseudotyping to examine spinal cord transduction and cell-type specificity. A pilot experiment was carried out with the purpose of choosing the best fetal route of injection showing widespread gene delivery in the spinal cord. Intraspinal injection showed robust e*GFP* expression along the entire spinal cord, while intracranial injection resulted in strong expression in the brain. Intravenous injection showed e*GFP* expression only in the liver. It has been previously demonstrated that stereotactic injections into adult rat or mouse spinal cord leads to limited spread from the site of injection [19]. However, in the current study widespread transduction of neurons along the entire spinal cord was seen following *in utero* administration. The volume of vector that was injected is enough to bathe the entire cord at this early stage, which may explain the efficient vector spread. This efficiency also suggests that the spinal pia membrane barrier to transduction observed in adult animal models [42] may not be such an obstacle at E16 in mice.

We evaluated a range of IDLV pseudotypes at the P10 time-point following intraspinal injection at E16: VSV-G, Rabies, RRV and Mokola. Immunostaining of spinal cord sections revealed a similar pattern, with very effective transduction of ChAT-positive motor neurons and DRG neurons, and lesser efficiency of transduction of NeuN neurons (11–36%, with Rabies being most efficient). No e*GFP* expression in astrocytes, oligodendrocytes or microglia was observed with any of the four vectors tested. Rabies IDLV also showed transduction of intercostal nerves which, together with DRG transduction, has been previously described following intraspinal injection of Equine Infectious Anemia Virus (EIAV) vector in fetal mice [18]. Effective and sustained e*GFP* expression in motor neurons and stable but lower levels of transduction of NeuN neurons were confirmed in mice analyzed 7 months after birth following treatment with VSV-G or Rabies IDLVs, which suggests stability of transgene expression in this model. The reasons for differential transduction proficiency of ChAT *versus* NeuN cells are currently unclear and may include accessibility at the E16 developmental stage, tropism, transduction efficiency and transgene promoter efficiency.

The transduction efficiency of ChAT-positive cells after intraspinal *in utero* delivery with IDLVs was remarkable, with apparently nearly all motor neurons (cervical, thoracic and lumbar) and DRG neurons showing eGFP fluorescence. The highest percentage of lower motor neuron transduction previously described in the literature was ~ 60%, with a single dose of AAV9 in P0/P1 mouse neonates [43]. The preferential neuronal tropism of the pseudotyped IDLVs (and standard integrating lentiviral vectors) was also observed in adult mouse and rat cord injections, but in these cases transduction was limited to the area around the injection site, motor neuron transduction levels were lower (up to c. 55% in mouse) and there was transgene expression in some astrocytes and oligodendrocytes [19]. The differences could be partly explained by the developmental stage as at E16 mouse neurogenesis is still on-going and the CNS during this period consists of a mixture of dividing, migrating, differentiating and differentiated neurons [44]. Additionally, there are relatively few astrocytes in the perinatal CNS, as astrogenesis in rodents occurs in the early post-natal period (the first two post-natal weeks) and the astrocytic end-feet are not fully developed [45]. Foust and co-workers demonstrated that AAV9 injection into neonatal mice at P1 mainly mediated neuronal transduction, while injections into adult mice showed more predominant glial transduction, even though this may at least partly depend on vector configuration [46].

Our comparison between IDLVs and scAAV9 showed significant differences in transduction efficiency and cell tropism following the same *in utero* intraspinal procedure. A number of factors may have had a role here, including differences in vector tropism and the promoter present in each vector (CMV in IDLVs, CAG in scAAV9). Although differences in virus titre are in favour of scAAV9, ChAT-positive neuron transduction was lower in mice injected with this vector, at about ~ 40%. *eGFP* expression by scAAV9 was also observed in non-neuronal cells such as GFAP-positive astrocytes. However, we did not observe any *eGFP* expression in oligodendrocytes. Staining of the spinal cord sections with anti-Iba-1 antibody did not reveal microglial activation upon *in utero* injection of any of the vectors.

In conclusion, *in utero* delivery of IDLVs represents a promising tool in studies of CNS biology and disease. The selective, extensive and highly efficient transduction of ChAT-positive dorsal root ganglia neurons and motor neurons observed creates opportunities for a number of novel studies. The key features of this approach include the early time point at which genes can be introduced into the nervous system, the selectivity for ChAT-positive cells and the relatively low number of IDLV particles required. Given the importance of motor neurons in neuromuscular disease, these findings may also have therapeutic implications for diseases such as spinal muscular atrophy.

## Appendix A Supplementary data

Supplementary material Image 2
