# Supplementary material for: High-efficiency transduction of spinal cord motor neurons by intrauterine delivery of integration-deficient lentiviral vectors
Source: J Control Release. 2018 Mar 10;273:99–107. doi: 10.1016/j.jconrel.2017.12.029 (PMC5845930; doi:10.1016/j.jconrel.2017.12.029)
Supplement: Supplementary file 1 — Supplementary material [file mmc1.docx]

**Supplementary Figures:**


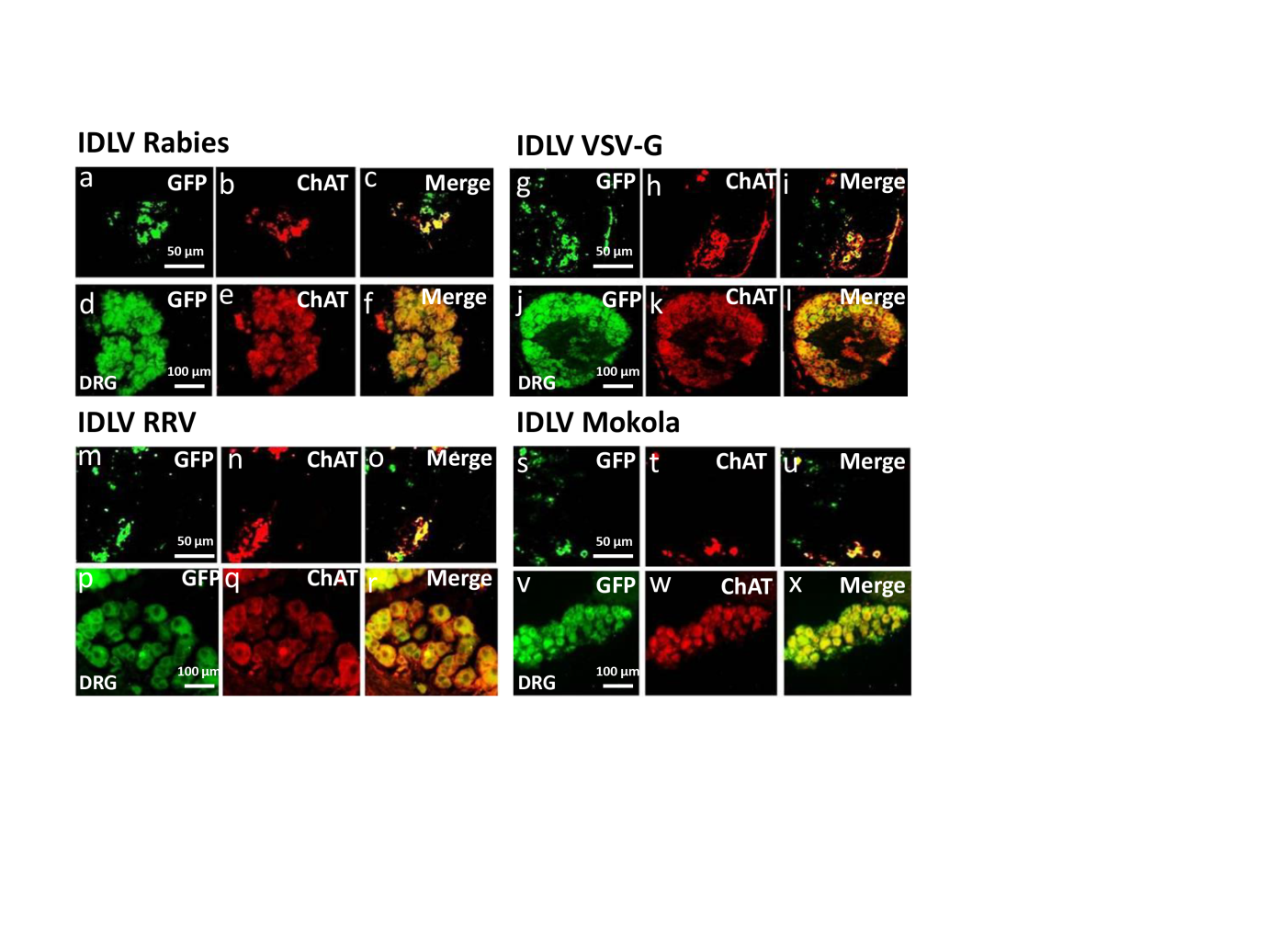


Supplementary Figure 1: Strong neuronal expression of *eGFP* in the thoracic segment of cords harvested at P10 following E16 intraspinal IDLV delivery. Transduction of thoracic motor neurons following intrauterine intraspinal delivery of the indicated IDLV CMV-eGFP vectors was assessed by co-staining with relevant markers. Strong transduction of ChAT+ motor neuron (a, b, c, g, h, i, m, n, o, s, t, u) and DRG neuron cell bodies (d, e, f, j, k, l, p, q, r, v, w, x) was observed.


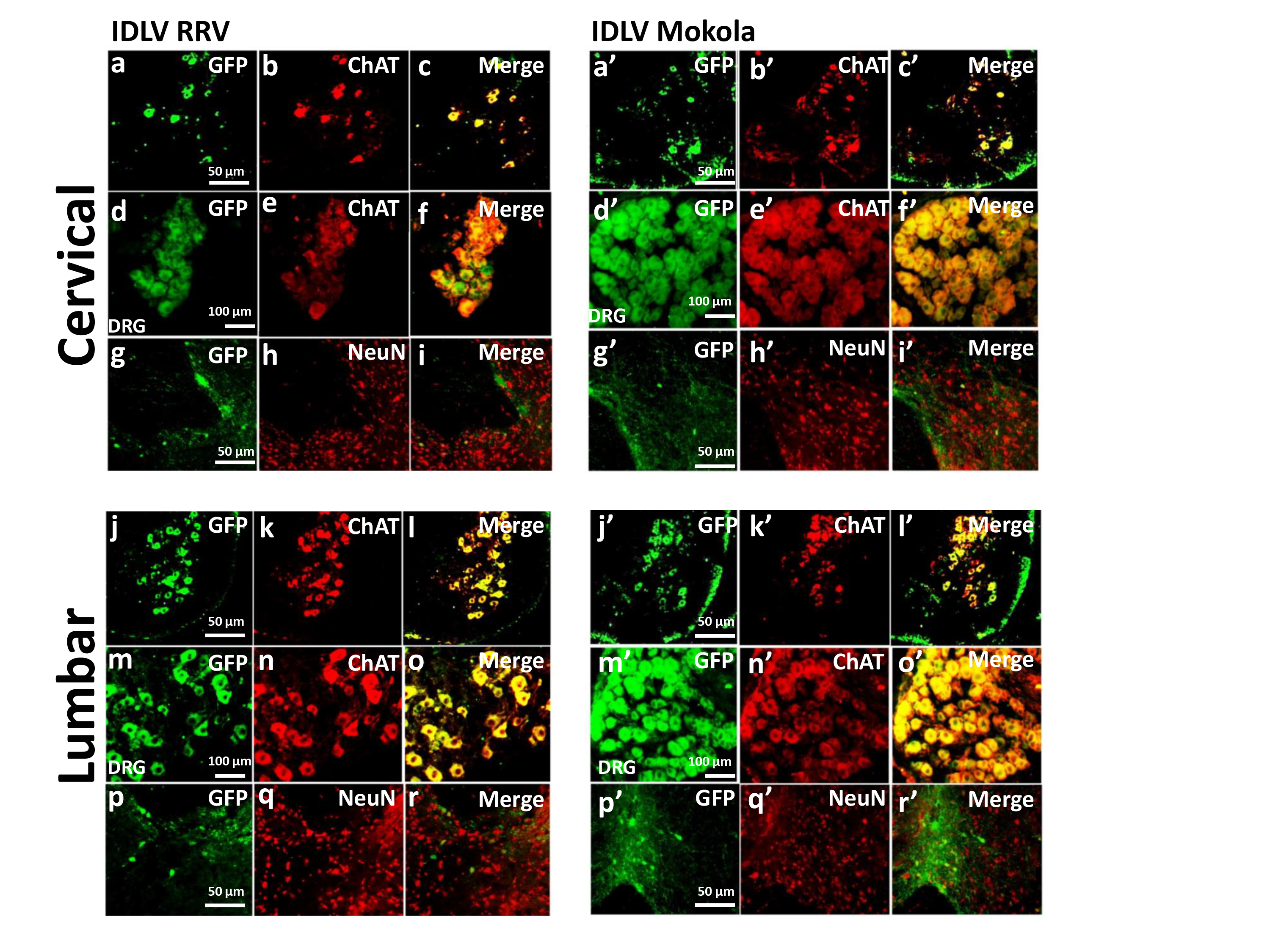


Supplementary Figure 2: Neuronal expression of *eGFP* after E16 intraspinal delivery of IDLV pseudotyped with RRV or Mokola envelope protein. Transduction of motor neurons and neurons was assessed by co-staining for ChAT or NeuN, respectively in cords harvested at P10. Efficient transduction of ChAT+ motor neuron (a, b, c, j, k, l, a’, b’, c’, j’, k’, l’) and DRG neurons (d, e, f, m, n, o, d’, e’, f’, m’, n’, o’) was noticed. *eGFP* expression was also visible in variable levels of NeuN neurons (g, h, i, p, q, r, g’, h’, i’, p’, q’, r’). Middle rows in all panels are DRGs.


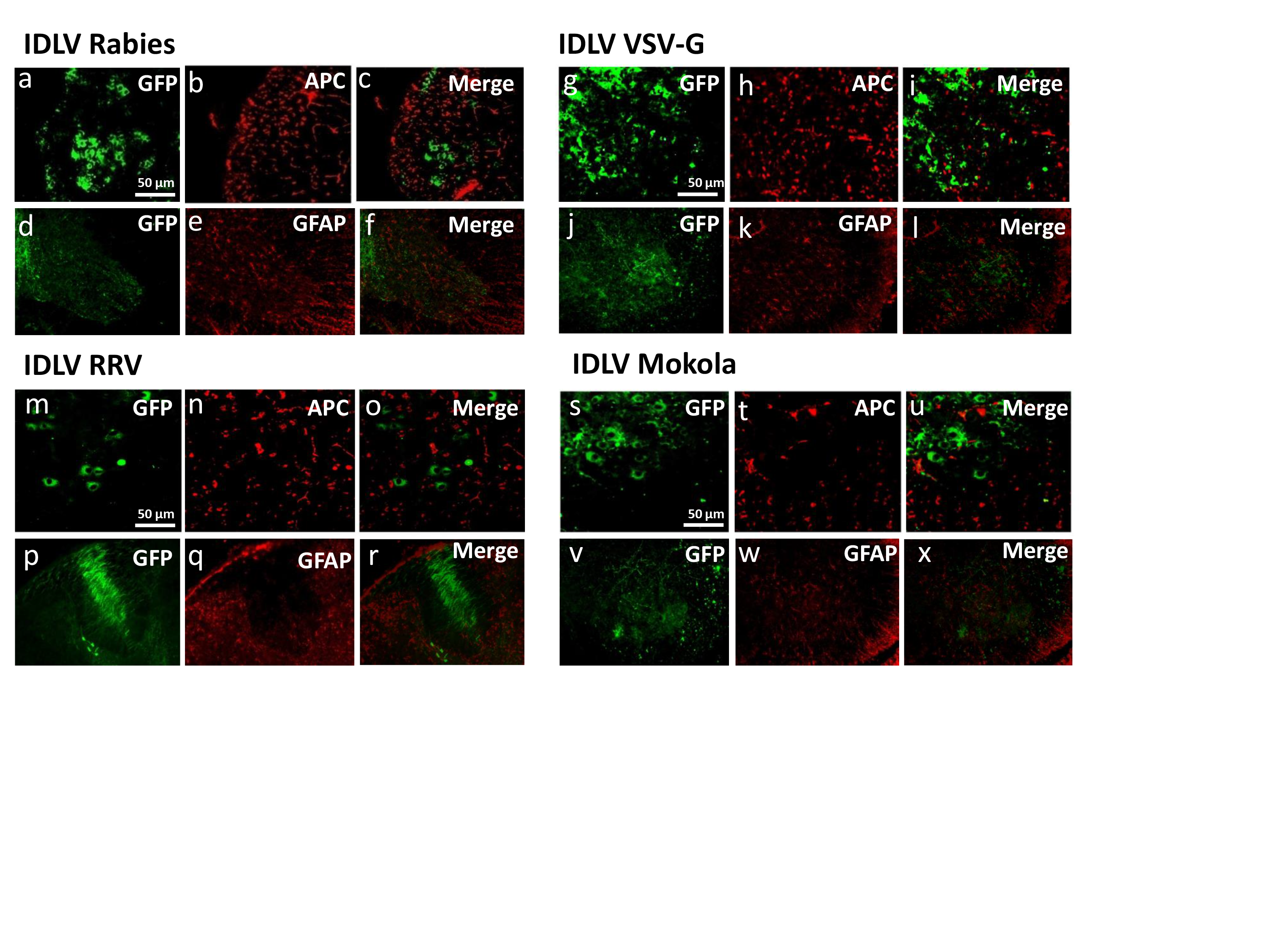


Supplementary Figure 3: Lack of transduction of oligodendrocytes and astrocytes by IDLVs following *in utero* intraspinal delivery. IDLVs expressing *eGFP* and pseudotyped with the indicated envelopes did not show significant transduction of APC immunoreactive oligodendrocytes (a, b, c, g, h, i, m, n, o, s, t, u) or GFAP-positive astrocytes (d, e, f, j, k, l, p, q, r, v, w, x) in cords harvested at P10.


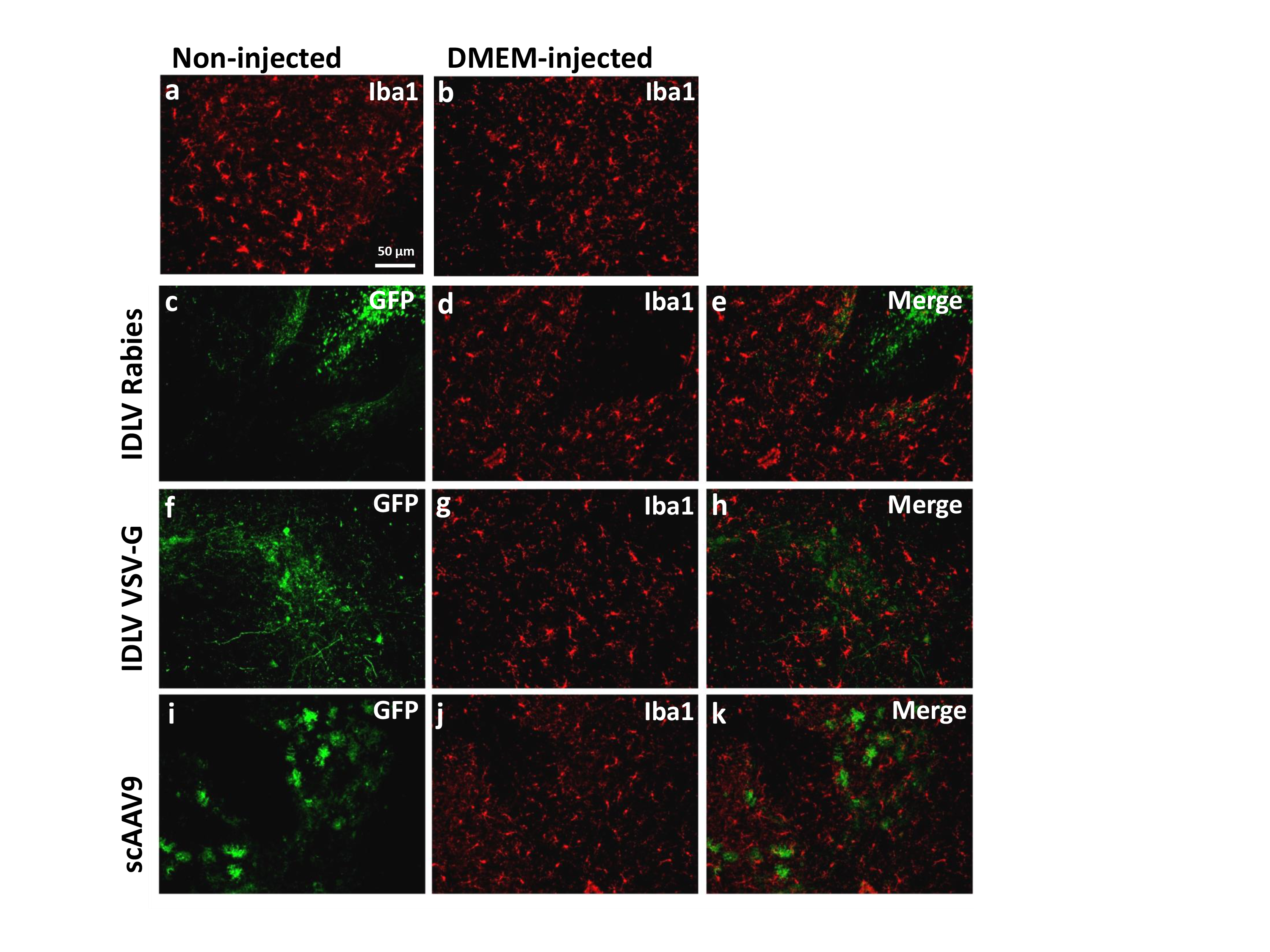


Supplementary Figure 4: Microglial staining of spinal cord sections injected with IDLV or scAAV9 did not show noticeable activation in cords harvested at P10. Staining of activated microglial cells was performed using Iba1 antibody. No obvious difference from those of non-injected or DMEM-injected mice was noticed.
